# Adapting the Healthy Eating Index 2010 for the Canadian Population: Evidence from the Canadian Community Health Survey

**DOI:** 10.3390/nu9080910

**Published:** 2017-08-21

**Authors:** Mahsa Jessri, Alena Praneet Ng, Mary R. L’Abbé

**Affiliations:** Department of Nutritional Sciences, Faculty of Medicine, University of Toronto, 150 College St., Toronto, ON M5S 3E2, Canada; m.jessri@mail.utoronto.ca (M.J.); alena.ng@mail.utoronto.ca (A.P.N.)

**Keywords:** Healthy Eating Index (HEI) 2010, Healthy Eating Index-Canada (HEI-C), Canada’s Food Guide, obesity, chronic diseases, Canadian Community Health Survey (CCHS), national nutrition survey

## Abstract

The Healthy Eating Index (HEI) is a diet quality index shown to be associated with reduced chronic disease risk. Older versions of the HEI have been adapted for Canadian populations; however, no Canadian modification of the Healthy Eating Index-2010 (HEI-2010) has been made. The aims of this study were: (a) to develop a Canadian adaptation of the HEI-2010 (i.e., Healthy Eating Index-Canada 2010 (HEI-C 2010)) by adapting the recommendations of the HEI-2010 to Canada’s Food Guide (CFG) 2007; (b) to evaluate the validity and reliability of the HEI-C 2010; and (c) to examine relationships between HEI-C 2010 scores with diet quality and the likelihood of being obese. Data from 12,805 participants (≥18 years) were obtained from the Canadian Community Health Survey Cycle 2.2. Weighted multivariate logistic regression was used to test the association between compliance to the HEI-C 2010 recommendations and the likelihood of being obese, adjusting for errors in self-reported dietary data. The total mean error-corrected HEI-C 2010 score was 50.85 ± 0.35 out of 100. Principal component analysis confirmed multidimensionality of the HEI-C 2010, while Cronbach’s α = 0.78 demonstrated internal reliability. Participants in the fourth quartile of the HEI-C 2010 with the healthiest diets were less likely to consume refined grains and empty calories and more likely to consume beneficial nutrients and foods (*p*-trend < 0.0001). Lower adherence to the index recommendations was inversely associated with the likelihood of being obese; this association strengthened after correction for measurement error (Odds Ratio: 1.41; 95% Confidence Interval: 1.17–1.71). Closer adherence to Canada’s Food Guide 2007 assessed through the HEI-C 2010 was associated with improved diet quality and reductions in the likelihood of obesity when energy intake and measurement errors were taken into account. Consideration of energy requirements and energy density in future updates of Canada’s Food Guide are important and necessary to ensure broader application and usability of dietary quality indexes developed based on this national nutrition guideline.

## 1. Introduction

In Canada, rates of adult obesity have increased from 6.1% in 1985 to 18.3% in 2011 [1]. The World Health Organization has identified diet as one of the most important risk factors contributing to the burden of chronic diseases worldwide [2,3,4], highlighting the importance of understanding the role diet plays in chronic disease prevention.

Diet quality indexes utilize a scoring system to assign points for adhering to a dietary pattern defined *a priori,* and can be used to measure the quality of diets within a population. The Healthy Eating Index (HEI) is a validated diet quality index first created in 1995 by the United States Department of Agriculture (USDA) to reflect the recommendations set out in the Dietary Guidelines for Americans (DGA) [5]. The DGA provides evidence-based dietary recommendations for Americans and focuses specifically on patterns of healthy eating to prevent chronic disease. The HEI-2010, modeled after the 2010 DGA recommendations, has been previously validated among the US population and has been shown to be associated with reduced risk of cancer [6,7,8,9], obesity [10,11], and mortality [12].

In Canada, modifications have been made to the 1995 and 2005 versions of the HEI for use among Canadian populations [13,14,15]. In 2009, Garriguet adapted HEI-2005 to Canada’s Food Guide (CFG) recommendations and applied the created index to a sample of respondents ≥2 years from the Canadian Community Health Survey (CCHS) Cycle 2.2 [15]. The mean total HEI-C 2005 score from this sample was 58.8 out of 100, suggesting that Canadian diet quality requires improvement [15]. To our knowledge, no adaptation of the HEI-2010 has been created for use among Canadians. Although the HEI-2015 (corresponding to the 2015 DGA) has been recently released, our objective was to create a Canadian adaptation of the HEI corresponding to the 2010 DGA, which was developed around the time as the current Canada’s Food Guide and includes updated scientific evidence in comparison to the 2005 DGA. The objectives of this study were therefore: (a) to develop the HEI-C 2010 by modifying the HEI-2010 recommendations to Canada’s national nutrition guideline, Canada’s Food Guide (CFG) 2007 [16]; (b) to evaluate the validity and reliability of HEI-C 2010; and (c) to examine the relationships between HEI-C 2010 scores and the diet quality of Canadians, as well as the association between index scores and the likelihood of being obese.

## 2. Materials and Methods

### 2.1. Data Source and Study Population

Data were taken from the Canadian Community Health Survey Cycle 2.2: Nutrition (CCHS 2.2), conducted from 2004 to 2005. Canadians across all provinces were sampled for CCHS 2.2; groups excluded constituted members of the Canadian Forces and individuals living on reserves, prisons, or long-term care facilities. The total sample size was 35,107 representing 98% of the country’s population after applicable survey weighting. Specific details regarding sampling design and data collection methods have been published elsewhere [17].

Exclusion criteria for our study included respondents <18 years of age, pregnant or lactating women and respondents with missing values for measured weight, height, physical activity or smoking status. The final analytical sample size for all analyses was 12,805.

### 2.2. Dietary Intake Assessment

Dietary intake was collected in-person by an initial 24-h dietary recall administered by a trained interviewer using a computerized modification of the Automated Multiple Pass Method [18]. Approximately 30% of the original respondents were invited to complete a second 24-h recall by phone. The Canadian Nutrient File supplement 2001b was used to obtain the nutrient and energy composition of reported foods [19].

To account for misreporting of energy intake, weight, height, and physical activity measurements were used to derive individual estimated energy requirements (EER) using Institute of Medicine (IOM) factorial equations [20]. Respondents’ reported energy intakes (EI) were compared to their EERs to identify energy under-reporters or over-reporters [21]. Under-reporters were defined as individuals whose EI:EER ratio was below 0.7 and over-reporters were defined as individuals with EI:EER ratios above 1.42 [21,22]. Those whose EI:EER ratios fell within 0.7–1.42 were considered plausible energy reporters [21,22].

### 2.3. Anthropometric and Lifestyle Measurements

Measurements of weight and height were collected at respondents’ homes using standard protocols [17]. Obesity was defined as a body mass index (BMI) ≥30 kg/m^2^. Interviewer-administered questionnaires were used to collect data on lifestyle and socio-demographic characteristics such as smoking status, alcohol intake, and physical activity level. Physical activity was defined using three possible activity levels (active, moderately active and inactive) based on respondents’ daily energy expenditure (DEE) collected from an interviewer-administered questionnaire. A respondent was considered physically active if their DEE was greater than 3, moderately active if their DEE was between 1.5 and 3, and inactive if their DEE was less than 1.5 [17].

### 2.4. Developing HEI-C 2010: Adapting HEI-2010 to CFG 2007

We developed the HEI-C 2010 using HEI-2010 scoring criteria [23], with adaptations for Canadian populations as described by Garriguet [15]. The HEI-2010 uses an energy density approach to control for energy intake by expressing servings per 1000 calories. To adapt HEI-2010 to CFG 2007, food intake was expressed as CFG 2007 servings corresponding to CFG 2007 recommendations based on respondent’s age and sex [16]. Similar to the HEI-2010, the scores for HEI-C 2010 ranged from 0 to 100, with higher scores representing better diet quality. The scoring criteria for the original HEI-2010 and the HEI-C 2010 can be found in supplementary materials (Tables S1 and S2, respectively).

HEI-2010 consists of 12 components: 9 adequacy components (features of a healthy diet that should be consumed in adequate amounts for optimal health) and 3 moderation components (features of a healthy diet that should be restricted for optimal health). While the HEI-2010 has separate components for total fruit and total vegetables, CFG 2007 combines fruits and vegetables into one food group. To adhere to this feature of CFG 2007, HEI-C 2010 included one combined component for total fruit and vegetables for a total of 8 adequacy components.

CFG 2007 emphasizes choosing vegetables and fruits more often than fruit juice, and to choose dark green and orange vegetables. Because orange vegetables are not included as a separate component in HEI-2010, this recommendation was not included in HEI-C 2010. Dark green vegetables correspond to the greens and beans component in HEI-2010. To reflect this emphasis on whole fruits and green vegetables in CFG 2007, the recommended servings for the components “whole fruit” and “greens and beans” in HEI-C 2010 were expressed as a percentage of the total fruit and vegetables recommendation in CFG 2007, using a method previously described [15]. In other words, because the maximum score criteria for the whole fruit component in HEI-2010 represented 21% of the sum of the maximum score criteria for total fruit and total vegetable components in HEI-2010, the Canadian adaptation of the whole fruit component was 21% of the serving recommendation for fruits and vegetables in CFG 2007. The maximum score criteria for the greens and beans component in HEI-C 2010 was determined similarly. Similar to HEI-2010, remaining servings of legumes in HEI-C 2010 were counted towards the greens and beans and total fruits and vegetables components only after allocation to the total protein foods and seafood and plant proteins components.

CFG 2007 recommends that 50% of grain products consumed be whole grain; thus the maximum score cut-off for the whole grains component in the HEI-C 2010 corresponded to half of the recommendations from the grain products food group in CFG 2007. Serving recommendations from the milk and alternatives and meat and alternatives food groups in CFG 2007 were used as the cut-off for maximum scores for the dairy and total protein foods components of the HEI-C 2010, respectively. The method described earlier for deriving maximum score criteria for whole fruits in HEI-C 2010 was used for the seafood and plant proteins component, with maximum score criteria expressed as a percentage of the meat and alternatives recommendations from CFG 2007. For all adequacy components, a minimum score of 0 was given if no servings were consumed; the only exception was the fatty acids component, where a ratio ≤1.2 for the sum of polyunsaturated fatty acids (PUFA) and monounsaturated fatty acids (MUFA) over saturated fat (SFA) ((PUFA+MUFA)/SFA) received a score of 0.

Similar to the original HEI-2010, moderation components of the HEI-C 2010 were reverse-scored due to the emphasis on restricting the consumption of these components (refined grains, sodium, and empty calories) in a healthy diet. Since CFG 2007 recommends that half of all grain products consumed should be whole grain, the minimum score cut-off for the refined grains component was set at ≥50% of grain products consumed as refined grains. For sodium, the IOM dietary reference intakes for sodium were used to assign minimum and maximum score cut-offs according to age and sex [20]. Following the methods used by Garriguet, respondents scored 10 points if their sodium consumption was at or less than the adequate intake (AI) for the appropriate age and sex group; 8 points if their sodium consumption was at the upper level (UL) intake; and 0 points if their consumption was twice the UL, with proportional scoring in between [15]. Scoring for the empty calories component was unchanged from the original HEI-2010. Empty calories included calories from solid fats, alcohol, and added sugars, the latter calculated using the method developed by Brisbois et al. as described by our group previously [24,25].

To compare diet quality among compliers, intermediate compliers, and poor compliers of CFG 2007, the scores for each HEI-C 2010 component were divided into quartiles. Those scoring in the top 25% were considered compliers for that component, those scoring in the bottom 25% were deemed poor compliers, and those scoring in the middle 50% were deemed intermediate compliers.

### 2.5. Statistical Analyses

All statistical analyses were performed using SAS (version 9.4; SAS Institute Inc., Cary, NC, USA) and were age and sex adjusted. Population survey weights provided by Statistics Canada were used to ensure representative population-level estimates. All standard errors, confidence intervals, and coefficients of variation were bootstrapped using the balanced repeated replication (BRR) technique with 500 replicates to account for the complex sampling design used in CCHS 2.2 [26].

Associations between index scores and intake of various foods and nutrients (as continuous variables) were calculated using weighted multivariate linear regression adjusted for age, sex, and energy intake (Model *a*) and age, sex, energy intake, and misreporting status (Model *b*). Weighted and bootstrapped logistic regression analyses were used to calculate odds ratios for the relationship between quartiles of calculated HEI-C 2010 scores and the likelihood of being obese, with quartile 4 of the index scores (representing healthiest diets) used as the reference group. Backwards stepwise regression was used to examine the effects of potential confounders in the model; only those with the greatest possible effect were included which were: age, sex, energy intake, physical activity, smoking status and misreporting status.

Weighted Pearson correlation coefficients were used to examine whether those with higher energy intakes were more likely to receive higher HEI-C 2010 scores by overconsuming calories. Weighted principal component analysis (PCA) was used to assess the multidimensionality of the HEI-C 2010 by examining whether any characteristics of the index contributed disproportionately to the total variation in scores [25]. Lastly, internal consistency (reliability) of the index was assessed by computing Cronbach’s coefficient α, with a criterion of α >0.7 demonstrating adequate internal consistency [27].

All analyses were initially performed using one day of dietary recall. To increase accuracy of findings by considering usual dietary intakes (long-term dietary exposure), the National Cancer Institute (NCI) method for estimating usual intakes was also used [28]. The NCI method requires one day of dietary recall from all respondents and a proportion of second day recalls to predict usual intake, accounting for random error (e.g., day-to-day variation), skewness of data, and correlations between dietary components, while adjusting for covariates of interest [28]. SAS macros for estimating usual dietary intakes using the NCI method are available online [29].

Using the NCI method and available second day dietary recalls, the usual intake-adjusted total mean HEI-C 2010 score and an unbiased slope estimate for the association between index scores and obesity were estimated [28,30]. The mean total HEI-C 2010 score was estimated adjusting for age, sex, weekend or weekday and sequence of dietary recall (first or second recall). The slope estimate for association with obesity was estimated with adjustments for age, sex, energy, physical activity, smoking, misreporting status and sequence of dietary recall. To avoid misclassification error, the usual intake-corrected HEI-C 2010 scores were entered into a logistic regression in continuous form to calculate odds ratios at the mid-value of each quartile (12.5, 37.5, 62.5, and 87.5) [31]. In order to adjust for energy in the slope estimation for the association between usual intake-corrected HEI-C 2010 scores and obesity, the usual consumption of energy also had to be estimated. This step was performed using SAS macros for bivariate models provided by the National Cancer Institute [29]. 

## 3. Results

**Figure 1:**
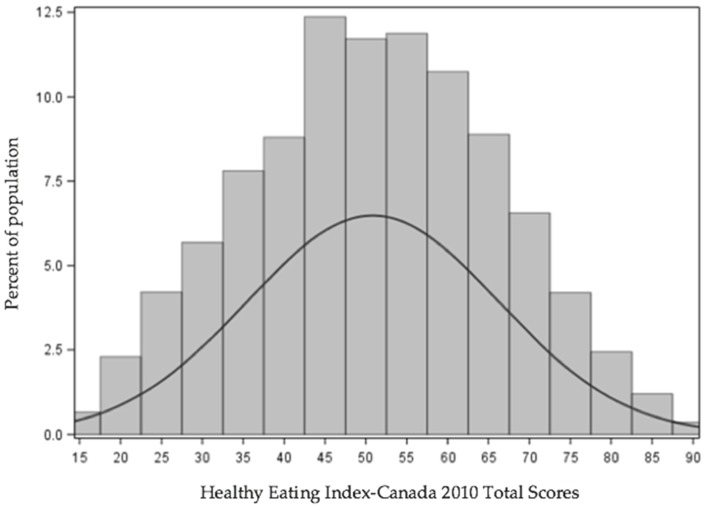
Weighted distribution of Healthy Eating Index-Canada 2010 scores among Canadian adults (*n* = 12,805). Results are for single day of recall. Data were trimmed according to Statistics Canada’s data release requirements.

Using one day of dietary recall, total HEI-C 2010 score was normally distributed (Figure 1). The mean total score was 50.84 ± 0.37 out of 100; after adjustment for covariates and measurement error correction using the NCI method, the mean total score was 50.85 ± 0.35 out of 100.

Face validity of the HEI-C 2010 was demonstrated by the associations between HEI-C 2010 scores and several lifestyle and socioeconomic characteristics (Table 1), which were consistently in the direction of the established hypothesis. Compared to quartile 1, participants with the healthiest diets in quartile 4 of the HEI-C 2010 score were more likely to be women (57.7% vs. 39.4%), older (49 years vs. 42 years) and multivitamin users (49.6% vs. 40.3%) (Table 1, all *p*-trends < 0.0001). Those with the highest HEI-C 2010 scores compared to the lowest were more likely to engage in positive lifestyle behaviors such as physical activity (21.7% vs. 13.6%) and abstain from smoking (57.3% vs. 30.2%), (all *p*-trends < 0.0001). Participants with the healthiest diets (quartile 4 of HEI-C 2010) were less likely to under-report energy intake (27.9% vs. 33.8%), however they were more likely to be energy over-reporters (12.5% vs. 9.8%, *p*-trend: 0.0146).

Higher HEI-C 2010 scores were significantly associated with greater consumption of beneficial micronutrients after adjusting for age, sex and energy intake (Table 2). Moving from quartile 1 to quartile 4 of the HEI-C 2010 score, consumption of fiber, polyunsaturated fatty acids and protein increased while consumption of added sugars, alcohol, and saturated fat decreased. There was no significant trend across quartiles for energy, total fat, or monounsaturated fatty acid intake. Similar results were observed when comparing intakes of the adequacy components and moderation components across quartiles of the HEI-C 2010 score (Table 3). Compared to quartile 1, those with the healthiest diets (quartile 4) were more likely to consume more fruits and vegetables (including whole fruit and dark green vegetables), protein (including seafood and plant proteins), whole grains and dairy. Those with a higher HEI-C 2010 score were also more likely to consume greater amounts of PUFA and MUFA compared to SFA. For moderation components, those with higher HEI-C 2010 scores were more likely to consume fewer empty calories (% energy) and refined grains (servings/day). All trends were significant (*p*-trend < 0.0001). Adjusting for misreporting did not change the significance or direction of the associations between HEI-C 2010 scores and nutrient or food group intakes (Model *b*, Table 2 and Table 3).

**Figure 2:**
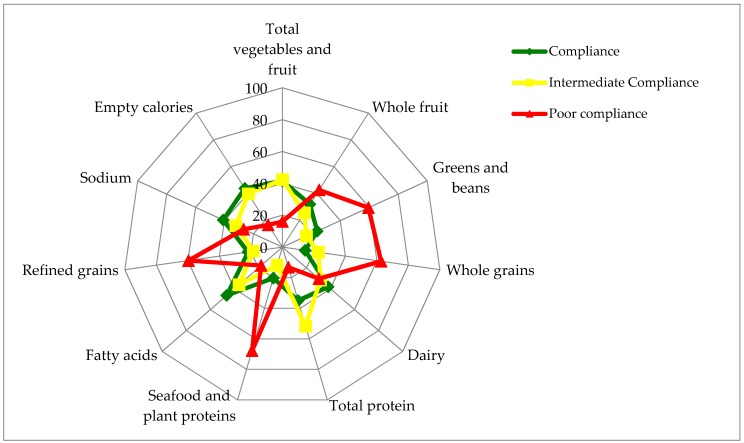
Weighted percentage of compliers (in green), intermediate compliers (in yellow), and poor compliers (in red) for each component of the Healthy Eating Index-Canada 2010 (HEI-C 2010) among Canadian adults (*n* = 12,805). Each spoke represents an individual HEI-C component. The outermost circle represents 100% prevalence, while the innermost circle represents 0% prevalence.

Figure 2 shows the prevalence of compliers, intermediate compliers, and poor compliers for each component of the HEI-C 2010. Full compliance to all index criteria was not observed. The greatest percentage of compliance was seen for the fatty acids component (46%), while the greatest percentage of poor compliance was observed for the seafood and plant proteins component (68%) (*p*-trend < 0.0001 for both). Additionally, poor compliance was observed most frequently for the whole grains (63%), refined grains (60%), greens and beans (59%), and whole fruit (43%) components (*p*-trend < 0.0001 for all).

The correlation between energy intake and HEI-C 2010 scores was examined to determine whether the HEI-C 2010 measures diet quality independent of energy intake (Table 4). The total HEI-C 2010 score had a non-significant low correlation with energy (*r* = −0.01, *p*: 0.2096). Low to medium correlations were observed between energy intake and component scores. One exception was the negative correlation between energy intake and the sodium component score (*r* = −0.67, *p* < 0.0001), indicating a positive association between energy intake and sodium consumption.

Principal component analysis was used to confirm the multidimensionality of the HEI-C 2010. Using a criterion of eigenvalue >1, four dimensions of the index were identified in the analysis which explained 63% of the total variance in HEI-C 2010 scores (Figure S1, Table S3). These results suggest that there was no one component of the HEI-C 2010 that contributed the majority of the variation in total scores. Internal reliability of the HEI-C 2010 was confirmed with a standardized Cronbach’s coefficient α of 0.78 (unstandardized: 0.80).

**Figure 3:**
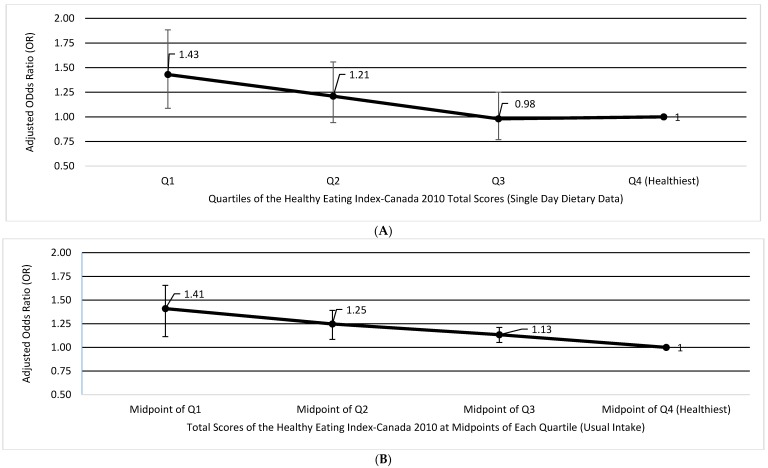
Weighted multivariate adjusted odds ratios (OR) and 95% confidence intervals (CI) representing the likelihood of being obese (BMI ≥30 kg/m^2^) for (**A**) quartile categories (Q) of HEI-C 2010 among Canadian adults (*n* = 12,805) using one day of dietary recall data with Quartile 4 as the reference, and (**B**) for estimated usual HEI-C 2010 scores derived from the NCI method, with estimated total scores entered as continuous. Four data points are shown (percentiles: 12.5, 37.5, 62.5, 87.5) corresponding to the midpoint of each quartile. Both models are adjusted for age, sex, energy, physical activity level, smoking status, and misreporting status. HEI-C: Healthy Eating Index-Canada; BMI: body mass index.

In our analyses of HEI-C 2010 and likelihood of obesity using a single day of dietary recall, those with lower scores had a higher likelihood of being obese (Odds Ratio (OR): 1.43; 95% Confidence Interval (CI): 1.09-1.89; *p*-trend: 0.0048) (Figure 3A). Only the association between quartile 1 vs. quartile 4 was statistically significant. Using the NCI method (for correction of random measurement error) with usual HEI-C 2010 scores entered as a continuous variable, the inverse association between HEI-C 2010 and obesity remained consistent and significant across all quartiles, with a 41% increased likelihood of being obese for those with the lowest quality diets (OR: 1.41; 95% CI: 1.17–1.71) (Figure 3B). It is notable that analyses without energy adjustments were not statistically significant (data not shown). 

Additional logistic regression models were examined to test the association between likelihood of obesity and quartiles of the HEI-C 2010 moderation sub-score, empty calories component sub-score and energy. Non-significant trends were observed between likelihood of obesity and the empty calories sub-score; a positive significant trend was observed between energy intake and likelihood of obesity, and between the moderation sub-score and likelihood of obesity (data not shown).

## 4. Discussion

To our knowledge, this is the first study to create the HEI-C 2010 by adapting Canada’s Food Guide recommendations to the 2010 version of the Healthy Eating Index for use among Canadian populations. We evaluated the validity and reliability of the developed HEI-C 2010 by applying it to a nationally-representative sample of Canadian adults in order to examine the associations between the index scores with diet quality and likelihood of obesity, while correcting for random and systematic measurement errors in self-reported data. Face validity of the HEI-C 2010 was confirmed by consistent relationships between the index score and lifestyle and socioeconomic characteristics in the hypothesized directions, while index reliability was confirmed by high Cronbach’s α. Our results suggest that adherence to the recommendations of the CFG 2007 (defined as higher HEI-C 2010 scores) was significantly associated with higher diet quality through greater intakes of beneficial nutrients and food groups, and lower intakes of dietary components to limit. While lower HEI-C 2010 sodium component scores (i.e., higher sodium consumption) was correlated with increased energy intake, this association between sodium and energy has been previously reported in the scientific literature [32,33].

Despite the more optimal diet quality among those who adhered greatest to the CFG 2007 recommendations, overall compliance to CFG 2007 recommendations was low. In this nationally-representative sample of Canadian adults, no respondent adhered to all recommendations of CFG 2007 (i.e., obtained a perfect total HEI-C 2010 score of 100/100). Poor compliance to index recommendations was most common for 5 out of 11 of the index components. Those who consumed the least healthy diets were more likely to be younger, male, physically inactive, and smokers. 

Our findings are consistent with those using the previous version of the HEI-C, HEI-C 2005. The mean total score for the HEI-C 2005 was 58.8 out of 100 in a previous Canadian sample of respondents ≥2 years of age [15]. This value is close to our reported mean error-corrected total HEI-C 2010 score of 50.85 out of 100, however the HEI-C 2005 mean score may be higher due to inclusion of children in the sample, as the diets of children tend to be of higher quality [34,35].

In the present study, the association between HEI-C 2010 scores and likelihood of obesity was moderately significant. Using the NCI method to correct for random measurement errors and to adjust for usual intake of energy strengthened this association, resulting in significant, inverse associations for the likelihood of being obese across quartiles. This is in contrast to our analyses on a single day of dietary recall, where only findings between quartile 1 and quartile 4 were statistically significant. An additional sensitivity analysis was performed whereby an intercept-only MIXED model was used to generate predicted values of HEI-C 2010 while adjusting for age, sex, smoking status, physical activity level and misreporting status. The predicted scores were then input into a logistic regression to examine their association with obesity. Results from this sensitivity analysis were similar to those derived from the NCI method, suggesting the NCI method’s ability to produce robust and reproducible estimates over analyses on single day dietary data (data not shown).

These results for the association between HEI-C 2010 and likelihood of obesity should be interpreted with caution, because the HEI-C 2010 does not consider energy intake in its scoring criteria. While the HEI-C 2010 includes considerations for energy-dense dietary components such as calories from alcohol, added sugars and solid fats in-line with the original HEI-2010 scoring, it differs from the original HEI-2010 in that it does not scale recommendations per 1000 calories (i.e., an energy density approach). This is a consequence of the age and sex-based recommendations in CFG 2007, which were developed mainly to aid Canadians in meeting their nutrient requirements and therefore do not consider individual energy requirements or physical activity level [36]. As a result, those who overconsume calories may be able to receive a higher HEI-C 2010 score, even though all current analyses on HEI-C 2010 were adjusted for energy intake. Our results confirmed that the largest frequency of energy over-reporters was among those with the highest HEI-C 2010 scores, a behaviour known to be associated with lower obesity rates, even though all analyses were adjusted for misreporting error [21]. 

While there are no studies investigating adherence to HEI-C in relation to obesity, a previous investigation by Bailey et al. using the original HEI-2010 demonstrated a positive association between higher total index scores and lower adiposity in a sample of US women [37]. In Canada, previous studies conducted by our group on CCHS 2.2 participants using energy-based diet quality indexes which include a penalty for energy overconsumption—such as the 2015 Dietary Guidelines for Americans Adherence Index (DGAI)—have demonstrated consistent inverse associations between closer adherence to index criteria and obesity risk [25,38]. The lack of consideration for energy requirements or physical activity levels in CFG 2007 is a potential limitation to the HEI-C 2010’s applicability as a nutrition surveillance tool in the context of healthy weight management or obesity prevention. This limitation was mitigated through statistical adjustments in this study, but may not be properly addressed in the context of population monitoring or obesity prevention programs.

A notable strength of our study includes adjustments for systematic measurement error (dietary misreporting) as well as random measurement error (day-to-day variation adjusted for using the NCI method) [21]. Previous studies have emphasized the need to adjust for systematic error in investigations of diet-disease relationships [21,22,39,40,41,42]. In addition, the NCI method has not been previously applied to a representative sample of Canadian adults for evaluating diet quality in relation to likelihood of obesity.

This study has a few limitations. First, the cross-sectional nature of CCHS 2.2 data prevents inferring causality. Second, CCHS 2.2 was conducted in 2004/2005 and Canadian dietary patterns may have changed since then; however, these analyses using CCHS 2.2 can be used as a baseline for comparing changes in diet quality using the newly-released CCHS 2015 data. Third, the statistical programs for multivariate models of the NCI method are developed for the HEI-2010, and currently are not available for multivariate analyses of other indexes. As a result, univariate and bivariate SAS macros were used, which would not completely remove random day-to-day variations in dietary intake. Despite this limitation, the use of the NCI method allows for more robust and accurate results compared to findings obtained from the use of single day data. We also performed additional sensitivity analyses to ensure plausibility of the findings from the final NCI models.

## 5. Conclusions

In summary, our findings suggest that closer adherence to Health Canada’s Canada Food Guide 2007 assessed through the HEI-C 2010 is associated with improved diet quality and moderate reductions in the likelihood of obesity when energy intake is considered in the analyses. In sensitivity analyses without adjustment for energy or consideration for random measurement errors, the association between HEI-C 2010 and obesity did not reach statistical significance. This, in part, reflects the nature of Health Canada’s 2007 Canada’s Food Guide to aid Canadians in meeting their nutrient recommendations regardless of overconsumption of foods, individual energy requirements or physical activity levels. In order to promote healthy eating patterns for weight management and prevention of obesity, future updates to Canada’s Food Guide should include recommendations for foods and beverages within an individual’s energy requirements and physical activity levels.

## Figures and Tables

**Table 1:** Weighted socio-demographic and lifestyle characteristics by quartile category of the HEI-C 2010 among Canadian adults (*n* = 12,805) ^1,2^.

	HEI-C 2010 Quartile Category				*p*-Trend ^3^
Characteristics	1 (Least Healthy)	2	3	4 (Healthiest)	
HEI-C 2010, range	<0.23	40.23–50.7	50.71–61.5	61.5≥	
HEI-C 2010 score, mean	31.22 ± 0.25	45.7 ± 0.1	55.9 ± 0.11	70.1 ± 0.23	<0.0001
Adequacy sub-score, mean	19.07 ± 0.31	26.04 ± 0.27	32.13 ± 0.26	40.5 ± 0.29	<0.0001
Moderation sub-score, mean	12.15 ± 0.26	19.66 ± 0.29	23.77 ± 0.25	29.6 ± 0.28	<0.0001
Female, %	39.37 ± 3.06	46.99 ± 2.88	56.2 ± 2.31	57.69 ± 3.46	<0.0001
Age, years	41.79 ± 0.45	45.63 ± 0.5	47.55 ± 0.49	49.01 ± 0.58	<0.0001
BMI, kg/m^2^	27.78 ± 0.21	27.64 ± 0.21	27.12 ± 0.25	27.06 ± 0.19	0.0451
BMI category, %					0.0104
Normal weight	34.48 ± 1.64	35.95 ± 1.77	39.05 ± 1.61	39.45 ± 1.48	
Obese	26.78 ± 1.53	25.54 ± 1.35	23.1 ± 1.42	22.8 ± 1.22	
Misreporting status, %					0.0146
Under-reporters of energy intake	33.75 ± 2.26	34.65 ± 2.3	31.7 ± 1.6	27.93 ± 1.79	
Over-reporters of energy intake	9.80 ± 1.04	9.44 ± 0.97	10.65 ± 0.8	12.48 ± 1.14	
Physical activity, %					<0.0001
Low-active	64.74 ± 1.53	62.12 ± 2.18	54.17 ± 1.95	51.02 ± 2.01	
Active	13.61 ± 0.91	15 ± 1.12	19.67 ± 1.23	21.74 ± 1.16	
Smoking status, %					<0.0001
Current daily smokers	33.64 ± 1.49	20.01 ± 1.23	17.74 ± 0.99	14.06 ± 1.11	
Never smoked	30.17 ± 1.64	46.55 ± 2.01	50.39 ± 1.69	57.25 ± 1.84	
Multivitamin users, %	40.29 ± 1.92	38.35 ± 1.77	46.22 ± 1.74	49.55 ± 1.9	<0.0001

^1^ Except for the HEI-C 2010 range, estimates are weighted means/percentages and bootstrapped variances (±SEM). ^2^ Values are adjusted for age and sex. Age is adjusted for sex only and sex adjusted for age only. ^3^ *p*-trend < 0.05 was considered statistically significant. HEI-C: Healthy Eating Index-Canada; BMI: body mass index; SEM: standard error of the mean.

**Table 2:** Weighted mean daily intakes of macro- and micronutrients by quartile category of the HEI-C 2010 among Canadian adults (*n* = 12,805) ^1,2^.

	Model	HEI-C 2010 Quartile Category				*p*-Trend ^3^
		1 (Unhealthy)	2	3	4 (Healthiest)	
HEI-C 2010 range		<40.23	40.23–50.7	50.71–61.5	61.5≥	
Energy intake, kcal/day	*a*	2024 ± 56	2037 ± 48	2106 ± 44	2175 ± 43	0.0665
	*b*	2401 ± 28	2431 ± 22	2462 ± 29	2450 ± 27	0.1641
Carbohydrate, % energy	*a*	47.91 ± 0.46	48.91 ± 0.51	49.49 ± 0.44	49.9 ± 0.47	0.0198
	*b*	47.36 ± 0.51	48.33 ± 0.47	49.01 ± 0.5	49.56 ± 0.55	0.0062
Fiber density, kcal/g	*a*	6.22 ± 0.12	7.83 ± 0.16	9.51 ± 0.19	11.68 ± 0.25	<0.0001
	*b*	6.01 ± 0.12	7.6 ± 0.15	9.35 ± 0.19	11.58 ± 0.26	<0.0001
Added sugar, % ^4^	*a*	13.66 ± 0.34	10.03 ± 0.31	8.39 ± 0.27	6.88 ± 0.23	<0.0001
	*b*	13.55 ± 0.35	9.93 ± 0.32	8.22 ± 0.29	6.68 ± 0.24	<0.0001
Total fat, % energy	*a*	31.86 ± 039	31.96 ± 0.46	31.25 ± 0.31	31.21 ± 0.36	0.4131
	*b*	32.36 ± 0.4	32.5 ± 0.43	31.66 ± 0.35	31.45 ± 0.39	0.1488
Saturated fat, % energy	*a*	11.67 ± 0.23	10.78 ± 0.19	9.75 ± 0.13	8.93 ± 0.12	<0.0001
	*b*	11.84 ± 0.22	10.96 ± 0.18	9.89 ± 0.14	9.01 ± 0.13	<0.0001
Monounsaturated fatty acids, % energy	*a*	12.41 ± 0.17	12.67 ± 0.23	12.58 ± 0.16	12.7 ± 0.2	0.633
	*b*	12.65 ± 0.17	12.94 ± 0.21	12.78 ± 0.18	12.82 ± 0.21	0.7105
Polyunsaturated fatty acids, % energy	*a*	4.87 ± 0.08	5.34 ± 0.1	5.76 ± 0.09	6.49 ± 0.12	<0.0001
	*b*	4.95 ± 0.1	5.24 ± 0.11	5.82 ± 0.1	6.52 ± 0.13	<0.0001
Protein, % energy	*a*	14.32 ± 0.21	16.32 ± 0.19	17.22 ± 0.29	17.66 ± 0.27	<0.0001
	*b*	14.29 ± 0.23	16.28 ± 0.21	17.25 ± 0.31	17.73 ± 0.29	<0.0001
Alcohol, % energy	*a*	5.91 ± 0.39	2.81 ± 0.22	2.03 ± 0.13	1.23 ± 0.1	<0.0001
	*b*	5.98 ± 0.43	2.89 ± 0.23	2.09 ± 0.17	1.26 ± 0.15	<0.0001
Cholesterol density, mg/1000 kcal	*a*	139.31 ± 4.03	150.34 ± 4.44	139.84 ± 4.6	128.66 ± 4.19	0.0044
	*b*	139.17 ± 4.06	150.04 ± 4.57	140.31 ± 4.71	129.59 ± 4.63	0.0074
Calcium density, mg/1000 kcal	*a*	377.18 ± 7.8	415.03 ± 6.97	436.63 ± 7.83	463.25 ± 7.86	<0.0001
	*b*	368.04 ± 7.75	405.42 ± 7.83	428.23 ± 8.1	456.98 ± 8.27	<0.0001
Vitamin A density in RAE, μg/1000 kcal	*a*	300.98 ± 10.56	348.54 ± 14.87	366.7 ± 10.68	448.83 ± 35.3	<0.0001
	*b*	291.82 ± 10.75	338.65 ± 15.5	359.35 ± 11.46	444.38 ± 31.59	<0.0001
Vitamin D density, μg/1000 kcal	*a*	2.29 ± 0.13	2.6 ± 0.08	2.98 ± 0.14	3.42 ± 0.17	<0.0001
	*b*	2.23 ± 0.16	2.64 ± 0.11	3.03 ± 0.18	3.46 ± 0.2	<0.0001
Vitamin C density, mg/1000 kcal	*a*	42.89 ± 1.8	59.35 ± 2.32	72.72 ± 2.43	82.56 ± 2.2	<0.0001
	*b*	40.01 ± 1.79	56.29 ± 2.16	70.23 ± 2.26	80.86 ± 2.3	<0.0001
Sodium density, g/1000 kcal	*a*	1588 ± 27	1594 ± 36	1560 ± 19	1448 ± 29	0.0012
	*b*	1576 ± 27	1580 ± 38	1554 ± 22	1449 ± 30	0.0026
Naturally-occurring folate density, μg/1000 kcal ^5^	*a*	93.36 ± 2.72	109.5 ± 2.56	129.95 ± 2.93	147.8 ± 4.03	<0.0001
	*b*	89.54 ± 2.42	105.39 ± 2.31	126.83 ± 2.88	145.86 ± 3.96	<0.0001
Folacin density from food sources, μg/1000 kcal ^6^	*a*	154.02 ± 3.41	176 ± 3.37	193.96 ± 3.22	198.06 ± 4.5	<0.0001
	*b*	150.63 ± 3.05	172.32 ± 3.06	191.27 ± 3.26	196.47 ± 4.72	<0.0001
Phosphorus density, mg/1000 kcal	*a*	565.97 ± 7.33	630.31 ± 6.09	685.43 ± 7.23	752.52 ± 8.83	<0.0001
	*b*	560.27 ± 7.43	623.95 ± 6.58	681.7 ± 7.75	751.17 ± 8.86	<0.0001
Magnesium density, mg/1000 kcal	*a*	137.24 ± 4.43	151.27 ± 1.72	173.57 ± 1.94	203.17 ± 3.21	<0.0001
	*b*	133.29 ± 3.97	146.95 ± 1.5	170.57 ± 1.89	201.53 ± 3.18	<0.0001
Iron density, mg/1000 kcal	*a*	6.21 ± 0.09	6.87 ± 0.08	7.29 ± 0.09	7.77 ± 0.1	<0.0001
	*b*	6.1 ± 0.09	6.75 ± 0.08	7.2 ± 0.09	7.72 ± 0.1	<0.0001
Zinc density, mg/1000 kcal	*a*	4.84 ± 0.08	5.5 ± 0.08	5.7 ± 0.08	6.124 ± 0.11	<0.0001
	*b*	4.81 ± 0.09	5.47 ± 0.09	5.7 ± 0.1	6.15 ± 0.12	<0.0001
Potassium density, mg/1000 kcal	*a*	1270 ± 17	1520 ± 28	1682 ± 19	1844 ± 24	<0.0001
	*b*	1236 ± 17	1482 ± 25	1656 ± 18	1831 ± 22	<0.0001
Caffeine density, mg/1000 kcal	*a*	166.19 ± 10.49	133.32 ± 5.08	124.91 ± 5.63	105.65 ± 4.83	<0.0001
	*b*	158.63 ± 9.77	124.93 ± 5.1	111.78 ± 5.53	103.56 ± 4.69	<0.0001

^1^ Except for the HEI-C 2010 range, estimates are weighted means/percentages and bootstrapped variances (±SEM). Estimates were determined using weighted multivariable linear regression. ^2^ Values are adjusted for age and sex (Model *a*) plus misreporting status (Model *b*). ^3^ *p*-trend was estimated using the HEI-C 2010 in its continuous form and represents the *p*-value associated with the linear regression coefficient. A *p*-trend < 0.05 was considered statistically significant. ^4^ Brisbois et al.’s method was used to derive estimates of added sugars [24,25]. ^5^ Naturally-occurring folate includes various forms of folate found naturally in foods. ^6^ Sum of quantities of naturally occurring folate in addition to folic acid without considering their differing bioavailability. HEI-C: Healthy Eating Index-Canada; SEM: standard error of the mean; RAE: retinol activity equivalents.

**Table 3:** Weighted mean of HEI-C 2010 components presented by quartile category of the HEI-C 2010 among Canadian adults (*n* = 12,805) ^1–3^.

	Model	HEI-C 2010 Quartile Category				*p*-Trend ^4^
		1 (Unhealthy)	2	3	4 (Healthiest)	
HEI-C 2010, range		<40.23	40.23–50.7	50.71–61.5	61.5≥	
Total fruits and vegetables, (servings/day)	*a*	3.08 ± 0.11	4.99 ± 0.17	6.1 ± 0.12	7.28 ± 0.18	<0.0001
	*b*	3.21 ± 0.13	5.13 ± 0.19	6.24 ± 0.17	7.42 ± 0.19	<0.0001
Whole fruit, (servings/day)	*a*	0.46 ± 0.12	1.05 ± 0.12	1.56 ± 0.1	2.25 ± 0.14	<0.0001
	*b*	0.46 ± 0.12 ^5^	1.1 ± 0.29	1.6 ± 0.31	2.28 ± 0.31	<0.0001
Greens and beans, (servings/day)	*a*	0.17 ± 0.09	0.36 ± 0.08	0.64 ± 0.08	0.98 ± 0.07	<0.0001
	*b*	0.17 ± 0.09 ^5^	0.36 ± 0.13	0.65 ± 0.14	0.98 ± 0.15	<0.0001
Whole grains, (servings/day)	*a*	0.07 ± 0.02 ^6^	0.44 ± 0.14	0.95 ± 0.15	2.43 ± 0.12	<0.0001
	*b*	0.07 ± 0.02 ^6^	0.39 ± 0.06	0.9 ± 0.07	2.38 ± 0.09	<0.0001
Dairy, (servings/day)	*a*	1.32 ± 0.09	1.62 ± 0.09	1.67 ± 0.1	1.77 ± 0.09	<0.0001
	*b*	1.28 ± 0.22	1.57 ± 0.23	1.63 ± 0.24	1.73 ± 0.23	<0.0001
Total protein foods, (servings/day)	*a*	1.86 ± 0.11	2.24 ± 0.11	2.37 ± 0.11	2.56 ± 0.1	<0.0001
	*b*	1.89 ± 0.25	2.27 ± 0.25	2.4 ± 0.25	2.59 ± 0.26	<0.0001
Seafood and plant proteins, (servings/day)	*a*	0.15 ± 0.07	0.28 ± 0.08	0.54 ± 0.07	0.98 ± 0.07	<0.0001
	*b*	0.15 ± 0.07 ^5^	0.28 ± 0.08 ^5^	0.54 ± 0.23	0.98 ± 0.23	<0.0001
Fatty acids, ((PUFA + MUFA)/SFA)	*a*	1.71 ± 0.06	1.88 ± 0.06	2.15 ± 0.07	2.38 ± 0.08	<0.0001
	*b*	1.7 ± 0.13	1.87 ± 0.14	2.14 ± 0.14	2.37 ± 0.14	<0.0001
Refined grains, (servings/day)	*a*	5.48 ± 0.2	5.64 ± 0.22	5.26 ± 0.2	3.54 ± 0.2	<0.0001
	*b*	5.64 ± 0.14	5.82 ± 0.14	5.44 ± 0.15	3.7 ± 0.18	<0.0001
Sodium, (mg/day)	*a*	3119 ± 47	3109 ± 50	3149 ± 45	2870 ± 75	0.0159
	*b*	3109 ± 58	3098 ± 64	3139 ± 60	2861 ± 91	0.0162
Empty calories, (% energy)	*a*	32.57 ± 0.46	18.39 ± 0.37	12.88 ± 0.31	9.11 ± 0.28	<0.0001
	*b*	32.38 ± 0.48	18.2 ± 0.43	12.66 ± 0.39	8.94 ± 0.35	<0.0001

^1^ Except for the HEI-C 2010 range, estimates are weighted means/percentages and bootstrapped variances (±SEM). Estimates were determined using weighted multivariable linear regression. ^2^ Values are adjusted for age, sex and energy (Model *a*) plus misreporting status (Model *b*). ^3^ Servings correspond to Canada’s Food Guide 2007 servings. ^4^ *p*-trend was estimated using the HEI-C 2010 in its continuous form and represents the *p*-value associated with the linear regression coefficient. A *p*-trend < 0.05 was considered statistically significant. ^5^ Estimate is the same as Model *a* due to low power. ^6^ Estimate is adjusted for age and sex only due to low power. HEI-C 2010: Healthy Eating Index-Canada 2010; SEM: standard error of the mean; PUFA: polyunsaturated fatty acids; MUFA: monounsaturated fatty acids; SFA: saturated fat.

**Table 4:** Weighted correlations among the total and component scores of the HEI-C 2010 as well as energy intakes among Canadian adults (*n* = 12,805) ^1^.

Components	Total Fruits and Vegetables	Whole Fruit	Greens and Beans	Whole Grains	Dairy	Total Protein Foods	Seafood and Plant Proteins	Refined Grains	Sodium	Empty Calories	Fatty Acids	Total HEI-C Score	Adequacy Score	Moderation Score	Energy
Total fruits and vegetables	1.00														
Whole fruit	0.49 *	1.00													
Greens and beans	0.27 *	0.15 *	1.00												
Whole grains	0.10 *	0.16 *	0.04 *	1.00											
Dairy	0.07 *	0.08 *	0.00	0.07 *	1.00										
Total protein foods	0.15 *	0.01	0.22 *	0.01	0.02	1.00									
Seafood and plant proteins	0.10 *	0.13 *	0.35 *	0.09 *	−0.04	0.33 *	1.00								
Refined grains	0.08 *	0.13 *	0.04 *	0.84 *	0.01	−0.01	0.10 *	1.00							
Sodium	−0.19 *	0.01	−0.08 *	−0.07 *	−0.29 *	−0.32	−0.06 *	0.10 *	1.00						
Empty calories	0.25 *	0.23 *	0.13 *	0.16 *	0.12 *	0.09 *	0.12 *	0.13 *	0.02 *	1.00					
Fatty acids	0.08 *	0.03 *	0.13 *	0.08 *	−0.35 *	0.13 *	0.26 *	0.05 *	0.00	0.12 *	1.00				
Total HEI-C score	0.49 *	0.48 *	0.37 *	0.59 *	0.19 *	0.21 *	0.39 *	0.59 *	0.09 *	0.68 *	0.31 *	1.00			
Adequacy score	0.62 *	0.53 *	0.49 *	0.50 *	0.32 *	0.38 *	0.50 *	0.39 *	−0.27 *	0.34 *	0.37 *	0.83 *	1.00		
Moderation score	0.14 *	0.22 *	0.07 *	0.46 *	−0.03 *	−0.07 *	0.10 *	0.57 *	0.46 *	0.78 *	0.11 *	0.78 *	0.30 *	1.00	
Energy	0.26 *	0.04 *	0.10 *	0.06 *	0.34 *	0.39 *	0.09 *	−0.12 *	−0.67 *	−0.13 *	0.03 *	−0.01	0.36 *	−0.42 *	1.00

* Statistically significant (*p* < 0.05). ^1^ Estimates are weighted Pearson correlation coefficients. HEI-C 2010: Healthy Eating Index-Canada 2010.

## Supplementary Materials

The followings are available online at www.mdpi.com/2072-6643/9/8/910/s1; Table S1: Scoring criteria for the Healthy Eating Index-2010 (HEI-2010). Table S2: Scoring criteria for the Healthy Eating Index-Canada 2010 (HEI-C 2010), a Canadian modification to the HEI-2010. Figure S1: Eigenvalues of the correlation matrix and scree plot from weighted principal component analysis of the Healthy Eating Index-Canada 2010 components showing the percentage of explained variance by each of the principal component dimensions among Canadian adults (*n* = 12,805). Table S3: Eigenvalues of the correlation matrix.
