# Supplementary material for: Adapting the Healthy Eating Index 2010 for the Canadian Population: Evidence from the Canadian Community Health Survey
Source: Nutrients. 2017 Aug 21;9(8):910. doi: 10.3390/nu9080910 (PMC5579703; doi:10.3390/nu9080910)
Supplement: Supplementary file 1 [file nutrients-09-00910-s001.docx]

Supplementary Materials: Adapting the Healthy Eating Index 2010 for the Canadian population: evidence from the Canadian Community Health Survey

Mahsa Jessri, Alena Praneet Ng and Mary R. L’Abbé

**Table S1.** Scoring criteria for the Healthy Eating Index-2010 (HEI-2010)^1^

| **Component** | **Max pts** | **Standard for max score** | **Standard for min score (0)** |
| --- | --- | --- | --- |
| Adequacy Sub-score | 60 |  |  |
| Total fruit | 5 | ≥ 0.8 cup eq./1000kcal | No servings |
| Whole fruit | 5 | ≥ 0.4 cup eq./1000kcal | No servings |
| Total vegetables | 5 | ≥ 1.1 cup eq./1000kcal | No servings |
| Greens and beans | 5 | ≥ 0.2 cup eq./1000kcal | No servings |
| Whole grains | 10 | ≥ 1.5 oz eq./1000kcal | No servings |
| Dairy | 10 | ≥ 1.3 cup eq./1000kcal | No servings |
| Total protein foods | 5 | ≥ 2.5 oz eq./1000kcal | No servings |
| Seafood and plant proteins | 5 | ≥ 0.8 oz eq./1000kcal | No servings |
| Fatty acids | 10 | (PUFA + MUFA)/SFA ≥ 2.5 | (PUFA + MUFA)/SFA ≤ 1.2 |
| Moderation Sub-score | 40 |  |  |
| Refined grains | 10 | ≤ 1.8 oz eq./1000kcal | ≥ 4.3 oz eq./1000kcal |
| Sodium | 10 | ≤ 1.1 g/1000kcal | ≥ 2.0 g/1000kcal |
| Empty calories | 20 | ≤ 19% of energy | ≥ 50% of energy |
| Total HEI-2010 Score | 100 |  |  |

^1^Specific details for the scoring of each component has been published previously [23].

**Table S2.** Scoring criteria for the Healthy Eating Index-Canada 2010 (HEI-C 2010), a Canadian modification to the HEI-2010^1,2^

| **Component** | **Max pts** | **Standard for max score** | **Standard for min score (0)** |
| --- | --- | --- | --- |
| Adequacy Sub-score | 60 |  | |
| Total fruits and vegetables^3^ | 10 | 4-10 servings | No servings |
| Whole fruit^4,^ **^5^** | 5 | 0.84-2.1 servings | No servings |
| Greens and beans^5^ | 5 | 0.42-1.05 servings | No servings |
| Whole grains^6^ | 10 | 1.5-4 servings | No servings |
| Dairy | 10 | 2-4 servings | No servings |
| Total protein foods | 5 | 1-3 servings | No servings |
| Seafood and plant proteins^7^ | 5 | 0.32-0.96 servings | No servings |
| Fatty acids | 10 | (PUFA + MUFA)/SFA ≥ 2.5 | (PUFA + MUFA)/SFA ≤ 1.2 |
| Moderation Sub-score | 40 |  | |
| Refined grains^8^ | 10 | <50% of grains refined | ≥50% of grains refined |
| Sodium^9^ | 8 to 10 | AI to UL | 2x UL |
| Empty calories^10^ | 20 | ≤ 19% of energy | ≥ 50% of energy |
| Total HEI-C 2010 Score | 100 |  | |

^1^Scoring criteria for the HEI-2010 was converted to servings based on the age and sex-specific recommendations found in CFG 2007. ^2^Scores between the maximum and minimum were assigned proportionally. ^3^Includes fruit juice. ^4^Excludes fruit juice. ^5^The standard for max scores of the “whole fruit” and “greens and beans” components represent 21% of the “vegetables and fruit” recommendation in CFG. ^6^The standard for max score of the “whole grains” component is 50% of the “grain products” recommendation in CFG. ^7^The standard for max score of the “seafood and plant proteins” component represents 32% of the “meat and alternatives” recommendation in CFG. ^8^The minimum score standard for the refined grains component is ≥50% of grain products consumed as refined grains based on CFG recommendations. ^9^Respondents scored 10 points if their sodium consumption was at or less than their adequate intake, 8 points if their sodium consumption was at their upper intake level and 0 points if their consumption was twice their upper intake level. ^10^Includes calories from solid fats, alcohol and added sugars.


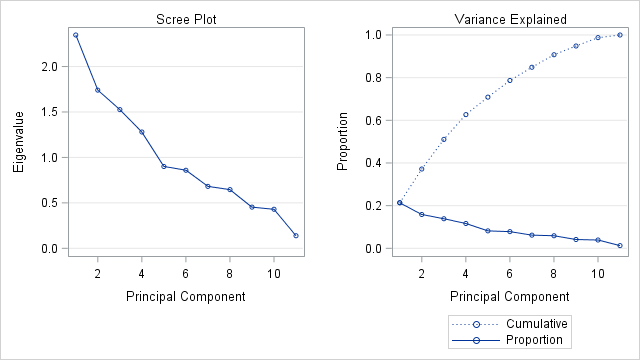


**Figure S1.** Eigenvalues of the correlation matrix and scree plot from weighted principal component analysis of the Healthy Eating Index-Canada 2010 components showing the percentage of explained variance by each of the principal component dimensions among Canadian adults (n = 12,805).

**Table S3.** Eigenvalues of the correlation matrix.

| **Principal Components** | **Eigenvalue** | **Proportion^1^** | **Cumulative^2^** |
| --- | --- | --- | --- |
| 1 | 2.34724176 | 0.2134 | 0.2134 |
| 2 | 1.74092820 | 0.1583 | 0.3717 |
| 3 | 1.52589066 | 0.1387 | 0.5104 |
| 4 | 1.27911804 | 0.1163 | 0.6267 |
| 5 | 0.90049230 | 0.0819 | 0.7085 |
| 6 | 0.85929148 | 0.0781 | 0.7866 |
| 7 | 0.68156591 | 0.0620 | 0.8486 |
| 8 | 0.64527780 | 0.0587 | 0.9073 |
| 9 | 0.45263453 | 0.0411 | 0.9484 |
| 10 | 0.42959515 | 0.0391 | 0.9875 |
| 11 | 0.13796416 | 0.0125 | 1.0000 |

^1^Proportion of the variance in Healthy Eating Index-Canada 2010 scores accounted for by each principal component. ^2^Cumulative proportion of variance in Healthy Eating Index-Canada 2010 scores accounted for by principal components.
